# Scrofula

**Published:** 1894-02

**Authors:** 


### SCROFULA.

[Proceedings of I. H. A.]

### Discussion upon Dr. J. H. Allen's Paper on "Scrofula."

Dr. Hoyne—The only criticism I have to make on the paper is that the author takes a long time to tell us what is already very well known, namely, that scrofula and syphilis have many points of resemblance, and sometimes seem almost identically the same thing. The father with tertiary syphilis communicates scrofula, not syphilis, to his children, and from this arises the fact that these scrofulous persons when they get a chancre get a mild one. When in our practice we come across a case of primary syphilis, if we have had much experience we recognize this fact. When a person not at all scrofulous contracts a chancre, he is very apt to go through the secondary and tertiary stages. Not so with a scrofulous person, for then we can succeed in cutting the disease short, or in curing it with great success. Moreover, I do not think urticaria can be traced to syphilis, as the doctor claims. It is an intensely itching disease, and syphilitic eruptions seldom or never itch. When any eruption itches excessively you can make up your mind it is not syphilitic.

Another point is that syphilitic eruptions are symmetrical or strongly inclined to symmetry, and urticaria is not so especially.

Dr. J. H. Allen—What I meant was that urticaria was a latent form of inherited syphilis. The fact that its aggravations and ameliorations are very much like those of Syphilinum, and that I have cured a great many cases with Syphilinum, leads me to think that it is of syphilitic origin. I have relieved these cases many times with ordinary remedies, but the attacks would return over and over again. When I prescribed Syphilinum the attacks stayed away.

Dr. F. O. Pease—I should like to ask the question whether, when the doctor has cured a certain case with Syphilinum, he is justified in saying that the case had a syphilitic basis, any more than when he cures a case with Chininum-sulphuricum he has a right to say that the patient has been poisoned with Quinine. Is it true because a certain nosode cures a case that the original of that nosode is present as the basis of the symptoms cured? Does it not lead us astray to refer all diseases to miasms, or to be always on the watch for miasms? Are there not diseases, and plenty of them, that do not have a miasmatic origin?

Dr. A. R. Morgan—The drift of this paper, and the use of nosodes unproved, is right away from principles which have been inculcated in *The Organon*. It is treating the disease instead of treating the sick man, and this is something we are expressly taught to avoid. It is a kind of empiricism which entirely does away with the necessity of studying our materia medica. Syphilinum may be an antidote to hives, but my experience coincides with that of Dr. Hoyne's, namely, that syphilitic eruptions do not itch. In the country we find hives most prevalent among the working farmers, a class of people among whom syphilis is not at all prevalent. Therefore, I think the doctor's claim that hives are syphilitic is unfounded and jumping to a conclusion which we cannot adopt.

Dr. Waddell—One would judge from Dr. Allen's paper that syphilis was the only cause of scrofula. If you take two children of the same family and with the same blood running in their veins, and bring one up on poor food, in a dark, damp cellar, and on impure air, and the other in healthful surroundings, you would have two very different children after a year or two. One would present many of the symptoms called scrofulous which the other would be free from. You might then change the conditions for the sickly one, but you could never quite restore its health with good surroundings. I believe there are other causes for scrofula than syphilis.

Dr. Hoyne—I did not mean to convey the impression in what I said that every case of scrofula was due to syphilis, but the large majority of cases of scrofula are syphilitic by heredity from syphilitic parents.

Dr. H. C. Allen—I think that many of us are very apt—too apt—when we cure a case with Syphilinum, to look upon it as establishing the fact that the case was hereditary syphilis in some form, and when Medorrhinum cures a case to look upon it as a case of sycosis in some form. This is a mistake and unjustifiable. Hahnemann says that Psorinum is not an isopathic remedy. He said there is no such thing as idem, but only similia. He said that Psorinum is as much changed by dynamization as gold is or any other substance, inert in crude form, and powerful when potentized. When we get a proving of a nosode it changes its character entirely, and may show it not adapted to cases similar from which it was taken.

Dr. J. H. Allen—I had not the slightest intention of offering my conclusions as final. I offer them as a contribution toward the clearing up of a difficult subject. We are too apt to call everything psoric. There are other miasms than psora, and we often mistake them for psora. In cases of urticaria in children you will always find the serrated or saw teeth; you also get soft, crumbling teeth, carious as soon as they are erupted. These are marked symptoms of syphilis; you get enlarged glands, with aggravation, in damp weather. These symptoms are highly suggestive of syphilis. I simply wanted to invite the members to study these cases from this point of view.

Dr. Holmes—It seems to me that right now is as good a time as any to get an understanding of these miasms. We are right in that line now. I think we will admit that scrofula and psora are two very indefinite terms. I have looked up their definitions several times in dictionaries, but did not get a clear, definite idea of their precise meanings. Psora was applied to any scaly eruption at one time, and scrofula is derived from sus, a sow, and was supposed to be due to pork eating. Professor Hoyne has just said that a child of a person afflicted with tertiary syphilis inherits not syphilis but scrofula. It seems to me that the second generation of a Jew will be a Jew still, and that syphilis will be syphilis through many generations. I do not understand why he says that not all cases of scrofula come from syphilis, and yet says that syphilis is the origin of scrofula. How can we say that this case came from syphilis and this case not?

Dr. Hoyne—You have just mentioned the fact that scrofula had been attributed to pork eating. Very few Jews are scrofulous, and they are almost exempt from syphilis. Probably the Jewish rite of circumcision has helped to prevent syphilis from spreading among them. In twenty-five years of experience among venereal patients, I have never seen but one Jew with syphilis, and he had a chancre upon the abdomen. All authorities agree that a man can transmit primary or secondary syphilis, but it cannot, as Dr. J. H. Allen says, skip a generation and come out in the following one. It comes out in the next succeeding generation as a form of hereditary syphilis, and it comes out on the skin during the early weeks of infancy. It does not wait until the child is eight or ten years old. In many cases the infant is covered with a rash when born. If it does not come out in four to six weeks after birth, it will not come out at all.

I remember one case, a woman with syphilitic psoriasis on the palms of the hand and the soles of the feet. She had been delivered of a syphilitic child, and was very anxious to have a healthy child; during the second pregnancy she was in my care. When the child was born the skin was just as healthy and smooth as could be, and it remained so ever afterward. She has since given birth to six children, all free from signs of syphilis.

Dr. J. H. Allen—I agree with the doctor as far as he has gone, but he is clinging to the old idea of primary, secondary, and tertiary stages. My paper goes a little further and endeavors to distinguish between the different miasms, which I do not believe are interchangeable. Syphilis must always be a form of itself; it cannot be scrofula.

Dr. E. E. Reininger—Bearing upon this point, we may notice the claim of the dominant school that the baccilli of scrofula are identical with those of tuberculosis.

